# No inflammatory effects after acute inhalation of barium sulfate particles in human volunteers

**DOI:** 10.1186/s12890-022-02021-y

**Published:** 2022-06-16

**Authors:** Christian Monsé, Götz Westphal, Monika Raulf, Birger Jettkant, Vera van Kampen, Benjamin Kendzia, Leonie Schürmeyer, Christoph Edzard Seifert, Eike-Maximilian Marek, Felicitas Wiegand, Nina Rosenkranz, Christopher Wegener, Rolf Merget, Thomas Brüning, Jürgen Bünger

**Affiliations:** Institute for Prevention and Occupational Medicine of the German Social Accident Insurance, Institute of the Ruhr University Bochum (IPA), Bürkle-de-la-Camp-Platz 1, 44789 Bochum, Germany

**Keywords:** Barium sulfate, Granular biopersitent particles, Human inhalation study, Induced sputum, Inflammatory markers, Particle-induced chemotaxis, Zinc oxide

## Abstract

**Background:**

Most threshold limit values are based on animal experiments. Often, the question remains whether these data reflect the situation in humans. As part of a series of investigations in our exposure lab, this study investigates whether the results on the inflammatory effects of particles that have been demonstrated in animal models can be confirmed in acute inhalation studies in humans. Such studies have not been conducted so far for barium sulfate particles (BaSO_4_), a substance with very low solubility and without known substance-specific toxicity. Previous inhalation studies with zinc oxide (ZnO), which has a substance-specific toxicity, have shown local and systemic inflammatory respones. The design of these human ZnO inhalation studies was adopted for BaSO_4_ to compare the effects of particles with known inflammatory activity and supposedly inert particles. For further comparison, in vitro investigations on inflammatory processes were carried out.

**Methods:**

Sixteen healthy volunteers were exposed to filtered air and BaSO_4_ particles (4.0 mg/m^3^) for two hours including one hour of ergometric cycling at moderate workload. Effect parameters were clinical signs, body temperature, and inflammatory markers in blood and induced sputum. In addition, particle-induced in vitro*-*chemotaxis of BaSO_4_ was investigated with regard to mode of action and differences between in vivo and in vitro effects.

**Results:**

No local or systemic clinical signs were observed after acute BaSO_4_ inhalation and, in contrast to our previous human exposure studies with ZnO, no elevated values of biomarkers of inflammation were measured after the challenge. The in vitro chemotaxis induced by BaSO_4_ particles was minimal and 15-fold lower compared to ZnO.

**Conclusion:**

The results of this study indicate that BaSO_4_ as a representative of granular biopersistent particles without specific toxicity does not induce inflammatory effects in humans after acute inhalation. Moreover, the in vitro data fit in with these in vivo results. Despite the careful and complex investigations, limitations must be admitted because the number of local effect parameters were limited and chronic toxicity could not be studied.

**Supplementary Information:**

The online version contains supplementary material available at 10.1186/s12890-022-02021-y.

## Background

Inhalation of granular biopersistent particles (GBP) can lead to fatal diseases such as lung cancer in rats in the case of chronic overload, even though GBP are only weakly toxic, as suggested by animal studies [1, 2]. It is generally assumed that this is primarily a consequence of chronic inflammation and that GBS in overload can also cause serious lung diseases in humans. Therefore, the threshold level value (TLV) for workplaces aims on the avoidance of inflammatory particle effects [1]. Based on this assumption, in Germany a threshold limit value of 0.3 mg/m^3^ was recommended for particles with a density of 1.0 g/cm^3^, mainly based on toxicological data of titanium dioxide [1]. The occupational exposure limit (OEL) for particles > 100 nm without substance-specific toxicity is based on this TLV [3] and was set at 1.25 mg/m^3^ for the respirable fraction and 10 mg/m^3^ for the inhalable fraction for particles with a density of 2.5 g/cm^3^. Particles with substance-specific toxicity are regulated with an OEL below 1.25 mg/m^3^.

Thus, barium sulfate (BaSO_4_) particles were used for ethical reasons in this study. BaSO_4_ is considered to be a chemically inert particle without substance-specific toxicity according to animal experiments [4, 5] and in vitro data [6, 7]. Although lysosomal solubility for BaSO_4_ was reported [8, 9], it is classified as GBP in Germany based on its water solubility. Aim of this study was to enhance the body of evidence for the assessment of GBP using an acute human inhalation study. Such human studies are rare and have not been done with BaSO_4_.

Sikkeland and co-workers conducted an inhalation study in humans using micro-sized aluminium oxide (volume median diameter particle size was 3.2 μm) as a representative of a chemical inert substance at concentrations between 3.8 and 4.0 mg/m^3^ for 2 h [10]. Elevated levels of neutrophils, protein level of IL-8 and gene signature related to several pathways were found in induced sputum collected 24 h after exposure, but no changes in blood parameters were measured 4 h after exposure. To our knowledge, further human inhalation- or epidemiological studies with BaSO_4_ are not available and it is not known whether micro-sized BaSO_4_ induces any toxic effects in humans after acute inhalation.

A controlled inhalation study with human volunteers at our exposure lab using nano-sized ZnO at concentrations up to 2 mg/m^3^ showed concentration-dependent inflammatory effects, including an increase of body temperature in several subjects, as well as an increase in blood neutrophils, and acute phase protein levels [11]. In an other ZnO study, subjects were exposed to nano- and micro-sized ZnO for 2 h at concentration levels of 2 mg/m^3^ each [12]. We could show that biological effects were more pronounced after exposure of micro-sized ZnO particles.

ZnO is not an inert particle and is known to cause metal fume fever after inhalation. In this study, the same effect parameters as in the ZnO studies were used. We hypothesized that the biological effect markers that were observed after inhalation of ZnO are not enhanced after acute inhalation of a chemically inert substance. Based on this assumption, we used a higher concentration for BaSO_4_ (4.0 mg/m^3^) than for the ZnO studies. The justification of the dose selection is discussed in detail in the discussion. Due to organizational reasons and time constraints, we were only able to perform an acute inhalation study. In this study, the endpoints clinical signs, body temperature, and inflammatory markers in blood and in induced sputum were examined in the subjects.

Moreover, we investigated if in vitro data based on particle-induced chemotaxis reflect these in vivo outcomes. This study may help to bridge the gap between animal and human in vivo experiments.

## Materials and methods

Since this study makes comparisons with previously conducted ZnO studies, many methods were adopted, especially from the study by Monsé et al. [12], which are described again for clarity.

### Micro-sized BaSO_4_ particles

The design and technical setup of the human whole-body exposure unit at our institute was described previously in detail [13]. The unit allows the exposure of up to 4 subjets at the same time. A self-constructed nebulizer was installed in the air conditioning duct of the exposure unit equipped with a 7.0 L stirred tank and a self-priming two-substance nozzle (model 970, Düsen-Schlick GmbH, Untersiemau, Germany). A suspension of 18.0 g of purchased BaSO_4_ (for medical purposes, CAS No. 7727-43-7, Merck GmbH & Co. KG, Darmstadt, Germany) in 5.0 L of water (water purification system, model Milli-Q Advantage A 10, Merck KGaA, Darmstadt, Germany) was continuously nebulized with pressurized air at 3.0 bar. The BaSO_4_ was sieved for 5 min before use, using the < 100 μm fraction (Vibratory Sieve Shaker, model AS 200 control, Retsch GmbH, Haan, Germany) to exclude larger clumps in the stirred tank. The suspension was stirred during dosing (260 rounds per min). Two flow baffles were installed in the tank to ensure a turbulent flow. The metering of the BaSO_4_ was controlled via a pulse width modulation by means of a compressed air shutdown of the two-substance nozzle. The aerosol droplets of the sprayed suspension completely dried out during the flight into the exposure unit and released the desired BaSO_4_ particles.

Briefly, constant target concentrations of 4.0 mg/m^3^ micro-sized BaSO_4_ were planned. The homogeneity of the particle atmosphere was given by the use of a ceiling fan and the type of air supply and exhaust. It was confirmed in preliminary tests by determining the particle size distributions at different locations in the exposure unit (data not shown). Sham exposures (0 mg/m^3^ BaSO_4_) were performed with filtered and conditioned air. All exposure scenarios were carried out with an air exchange rate at 12 per hour (360 m^3^/h) with a room temperature of 23.4 °C (± 0.5 °C) and a relative humidity of 44.5% (± 1.8%).

### Characterization of BaSO_4_ particles

An Aerosol Particle Sizer (APS, model 3321, TSI Inc., Shoreview MN, USA, equipped with a 1:20 aerosol diluter, model 3302 A, TSI Inc.) measured the micro-sized particle numbers and size distributions every 5 min. (Additional file 1: Figure S1). The measurement system was installed directly in the exposure unit near the subjects to minimize particle losses to the instrument inlet. Mass concentration measurements of airborne BaSO_4_ were recorded at 1-min intervals using a tapered elemental oscillating microbalance (TEOM, model 1400a, Rupprecht and Patashnik, Albany NY, USA). Both the airborne mass of BaSO_4_ particles and the drying process of the BaSO_4_ suspension were confirmed by gravimetric measurements using a total dust sampling system (model GSP, GSA Messgerätebau GmbH, Ratingen, Germany) with cellulose nitrate filter (Sartorius Stedim Biotech GmbH, 8 µm pore size, 37 mm diameter). Two filters were exposed in sequence to a volume flow of 10.0 L/min for 96 and 69 min each (air sampler, model SG10-2, GSA Messgerätebau GmbH, Ratingen, Germany). The deviation between the TEOM and gravimetric measurements for the first filter was + 0.95% and for the second – 0.77%.

The specific surface area was determined using a BET device (BET, model Gemini VII 2390a, Micromeritics GmbH, Aachen, Germany). BaSO_4_ was dried at 300 °C for 60 min as a pretreatment, and a surface area of 3.2 m^2^/g was determined. A second measurement showed a slightly smaller surface area of 2.8 m^2^/g after further drying at 300 °C for 90 min. The measurements confirm that the used BaSO_4_ has no appreciable porosity and agree very well with measurements known from the literature [14].

The particles were taken directly from the chemical packaging, put on a scanning electron microscopy pin stub and characterized by scanning electron microscopy (SEM, model Zeiss Supra 40VP, Carl Zeiss Microscopy Deutschland GmbH, Oberkochen, Germany) with a nominal resolution of 2 nm. The micro-sized BaSO_4_ particles consisted of individual crystals and were rounded at their edges (Additional file 1: Figure S2).

An elemental analysis of the BaSO_4_ particles (Mikroanalytisches Labor Pascher, Remagen, Germany) showed a chemical purity of > 99% with regard to organic impurities. The measured carbon content was < 0.01% and the hydrogen content < 0.09%. To exclude the compound barium carbonate, which is soluble in the body and therefore toxic, the carbonate content was analyzed. It was negligible at < 0.01%.

### Participants

Sixteen healthy nonsmoking volunteers (8 women, 8 men) with a median age of 28 (range 20–37) years participated in the study (Table 1). The subjects reported no previous exposure to airborne BaSO_4_ and did not show bronchial hyperresponsiveness to methacholine as assessed with a reservoir method [15]. The study participants had to be able to produce sputum after induction with 0.9% saline according to our criteria (eosinophils < 1%, epithelial cells < 95% and neutrophils not dominant) in order to exclude subjects with airway inflammation and to make sure that the material originated from the lower airways. Standard baseline laboratory parameters were within normal ranges. Specific IgE antibodies (sIgE) to ubiquitous aeroallergens (atopy screen sx1, Phadiatop, ImmunoCAP system, ThermoFisher Scientific, Phadia AB, Uppsala, Sweden) as well as total IgE were measured with the ImmunoCAP 250 system. A positive atopic status was assumed in case of a sIgE concentration to sx1 > 0.35 kU_A_/L. Two atopic subjects (one woman, one man) without any clinical manifestation of allergies were included in the study cohort.

**Table 1:** Characteristics of the study subjects

Parameters	Total	Male	Female
	N = 16	N = 8	N = 8
Age [years]	28 (20–37)	28 (23–37)	24 (20–35)
Height [cm]	178 (160–194)	182 (175–194)	168 (160–190)
Weight [kg]	75 (50–122)	89 (64–122)	66 (50–95)
BMI [kg/m^2^]	24.9 (18.4–32.4)	26.3 (19.1–32.4)	22.6 (18.4–29.7)
Total IgE [kU/L]	23.1 (< 2–643)	19.4 (3.83–643)	26.1 (< 2–51.2)
sIgE to sx1 [kU_A_/L]	0.06 (0.03–5.71)	0.065 (0.03–0.75)	0.06 (0.04–5.71)
sIgE to sx1 > 0.35 kU_A_/L [n]	2	1	1

Medians and ranges are listed. BMI = body mass index. Specific IgE (sIgE) to sx1 ≥ 0.35 kU_A_/L is an indicator of sensitization to environmental allergens

### Study design

**Fig. 1:**
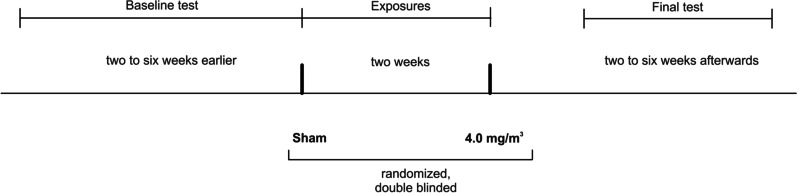
Time line of this study

16 subjects were exposed according to the scheme for 2 h (Fig. 1), with a minimum interval of two weeks between each exposure (sham and 4.0 mg/m^3^ BaSO_4_, subjects served as their own control). The subjects were at rest except for two periods of 30 min with moderate physical activity set to 15 L/min/m^2^ (corresponding to a median work load of 60 watts (range 43 to 90 watts)) on a cycle ergometer. The aim of this setting was to allow the subjects to inhale approx. 10 m^3^ in accordance with the assumption of the German MAK Commission for the respiratory volume of humans of 10 m^3^ per 8-h working day [1]. The retrospective evaluation resulted in an averaged value of 9.48 ± 1.44 m^3^ for the 16 subjects. Exposures were randomized and double blinded. Medical examinations were performed before, directly after and approximately 22 h after the start of exposure.

Baseline testing was performed before the first exposure. The following examinations were done: detailed questionnaire-based medical history, physical examination, blood sampling, sputum induction and analysis, lung function testing, cotinine measurement in urine, measurements of fractional exhaled nitric oxide (FeNO), electrocardiogram, blood pressure, spiroergometry to assess the work load, methacholine testing and body temperature. After the last exposure a final testing was performed including physical examination, blood and induced sputum sampling and analysis, electrocardiogram, blood pressure, lung function testing and body temperature. In addition, vital functions (electrocardiogram, blood pressure) were monitored during the exposures, which were always carried out from 10 to 12 am. Time of sample collection was recorded in order to adjust for possible diurnal varations in the biomarker levels.

### Questionnaire

All subjects answered a questionnaire addressing flu-like symptoms (at least one of three symptoms: feeling of fever, feeling sick and muscle pain) and airway irritation (throat irritation and/or cough) at different time points (before exposure, directly after and 22 h after exposure). To avoid any information bias we added questions about clearing throat, shortness of breath, fatigue, headache, feeling warm, discomfort, chills, and feeling unwell. All symptoms were graded according to severity (not at all (0 score point), barely (1 point), little (2 points), moderate (3 points), strong (4 points), very strong (5 points)). Sum scores and percentage of sum scores were generated for each study participant, described in detail previously [12].

### ECG and blood pressure

Portable PSG devices (SOMNOscreen™ plus, SOMNOmedics GmbH, Randersacker, Germany) were used with 2 canal ECG at 512 samples/s, SpO2 and Cuffless Continuous Blood Pressure Measurement based on puls transit time. Additionally, the blood pressure was manually measured before and after each exposure.

### Body temperature

Subjects measured their own body temperatures using a digital thermometer (model MT3001, Microlife AG, Widnau, Switzerland) before, during and after BaSO_4_ exposure and additionally every 2 h until the next day, but not during sleep. All participants were instructed to put the thermometer at minimum for 1 min under the tongue (sublingual) with the mouth closed and no drinking or eating 5 min prior to measurements. The limit value at T = 37.5 °C was considered a fever. The measurements were subject to a deviation of 0.1 °C. No separate quality assessments were carried out, the plausibility of the measurement results was assessed by expert judgment.

### Blood parameters

Blood samples (12 ml each) were obtained at the baseline examination, directly before exposures, 22 h post-exposures and at the final examination. Inflammatory markers (differential blood cell count, C-reactive protein (CRP), serum amyloide A (SAA)) were analyzed using standard methods. The total and differential blood cell counts were determined using the Coulter counter- method with UniCell DxH800 (Beckman Coulter Inc., Brea, CA, USA). ELISA techniques were used to quantify the following serum biomarker: SAA (Invitrogen™ Carlsbad, CA, USA; detection of human serum amyloid A1 cluster (Hu SAA) in the range of 9.4–600 ng/mL), and CRP (high sensitive ELISA from IBL International, Hamburg, Germany; range 0.4–19 µg/mL). The parameters in this study were examined using the same methods as described in [12].

Further standard clinical parameters of renal and liver function were determined during the recruiting process of the subjects. Determination of creatinine in urine was done via the Jaffé method (measuring range 0.3—25.0 mg/dL).

### Induced sputum

Sputum samples were obtained at the baseline examination, 22 h post-exposures and at the final examination, but not directly before exposures. This procedure eliminates the possibility that repeated sputum recovery within a short time period may induce inflammatory effects triggered by sputum induction itself. According to the procedure used in several studies [12, 16, 17] sputum induction was carried out by inhalation of nebulized isotonic saline solution (0.9% sodium chloride (NaCl); Pariboy, Pari GmbH, Weilheim, Germany) for 15 min. Concentrations of interleukin-8 (IL-8), matrix metalloproteinase-9 (MMP-9) and tissue inhibitors of metalloproteinases-1 (TIMP-1) were determined in the appropriate immunoassays based on monoclonal or polyclonal antibodies (Pharmingen, Heidelberg, Germany, Assay Design and/or Bio Vendor, all: Heidelberg, Germany) according to the recommendations of the manufacturers. The total protein determination was carried out with bovine serum albumin as a standard with a measuring range of 10 to 100 mg/L. The respective lower quantification limit was 3 pg/mL for IL-8, 31.2 pg/mL for MMP-9 and 9.76 pg/mL for TIMP-1 [18].

### FeNO

Increased excretion of nitric oxide (NO) in the airways is expected due to inflammatory processes. FeNO was measured using a portable electrochemical analyzer (NIOX Mino, Aerocrine, Solna, Sweden) taking into account the guidelines of the American Thoracic Society and European Respiratory Society [19].

### Lung function testing

Lung function was recorded using both body plethysmography [20] and spirometry [21] in a linked maneuver with a MasterLab (Vyaire Medical GmbH, Höchberg, Germany). A battery of different parameters was evaluated (e.g. airway resistance, lung volumes, and flows).

### Data analysis of effect parameters

Characteristics of subjects were expressed as medians as well as minimum and maximum (see Table 1). Descriptive analysis was performed for each variable stratified by exposure (sham, 4.0 mg/m^3^ BaSO_4_) and time of measurement (before, 22 h after exposure). Graphical representations were illustrated with boxplots (median and the 25–75% percentiles). Effects were compared between before and 22 h after exposure. Exposure groups were compared using paired Student`s t-test for normal or log-normal distributed variables. If normal distribution could not be assumed Wilcoxon signed-rank test was used.

The problem of multiple comparisons was counteracted using the Bonferroni correction [22]. Individual descriptive analyses were performed for body temperature with a cut-off of ≥ 37.5 °C. Differences in the blood and sputum parameters between sham and BaSO_4_ exposures and time of measurements were examined using multivariable generalized estimating equations (GEE) logistic regression [23]. Here, we compared the differences for each parameter separately for the time of measurement and exposure using odds ratios (OR) and 95% confidence intervals (CI). These analyses were performed taking into account the sample time and concentration.

### Statistical analysis

To represent the dose–response relation in the in vitro experiments more precisely, model fits like the four-parameter log-logistic model or the $E_{\max }$ model were used. According to O’Connell et al. [24] the four-parameter log-logistic model is for data following a sigmoidal shaped curve and defined for concentration $x$ as:

$$f\left( {x, \left( {b,c,d,e} \right)} \right) = c + \frac{d - c}{{1 + \exp \left( {b \left( {\log \left( x \right) - \log \left( e \right)} \right)} \right)}} ,$$

with b, c, d, and e as corresponding parameters. c and accordingly d indicate the lower respectively upper asymptote, b serves as parameter for the slope of the curve and e is the 50% effective concentration EC_50_. Furthermore on the report of Pinheiro et al. [25] the $E_{\max }$ model is defined as:

$$f\left( {x, \left( {E_{0} , E_{\max } ,EC_{50} } \right)} \right) = E_{0} + E_{\max } \frac{x}{{EC_{50} + x}} ,$$

with $E_{0}$ corresponding to the basal effect at placebo concentration x = 0, $E_{\max }$ representing the maximum change in effect and $EC_{50}$ being the 50% effective concentration.

### Estimation of lung deposition efficiency

To estimate the BaSO_4_ particle lung deposition efficiency we modified the open-source code [26] based on the International Committee for Radiological Protection (ICRP) Publication 66 [27]. Further information is given in [18].

### Particle induced cell migration assay

In order to assess whether ZnO and BaSO_4_ differ in their acute biological effects, we analyzed the chemotactic attraction of differentiated human leukemia cells (dHL-60 cells) in response to cell supernatants of particle- challenged NR8383 rat alveolar macrophages, as a model for the particle-induced accumulation of neutrophils in the inflamed lung [6]. The substances used in the in vitro experiments were the same as those used in the human experiments (aerodynamic diameter ZnO: 1,33 µm; aerodynamic diameter BaSO_4_: 1,90 µm). dHL-60 cells have properties similar to that of physiological neutrophil granulocytes. Briefly, the cell supernatants were used to investigate migration of dHL-60 cells. We challenged with compounds separately. As a positive control, a silica (SiO_2_) reference sample was used (CAS No. 7631-86-9, Lot MKBF2889V, 99.5%, 10–20 nm, Sigma-Aldrich, Steinheim, Germany). In order to calculate a continuous course of each treatment, dose–response models were fitted for each compound. Though four-parameter log-logistic models were used for ZnO and BaSO_4_ while SiO_2_ was modeled by a E_max_-model. The choise of the model is based on the known biological activity of the compound. In preliminary tests, for example, ZnO showed a plateau effect after a dose of 20 μg/cm^2^ and higher, which the fitted model maintains. A detailed description of the preliminary test can be found in the Additional file 1: Figure S4. Modelling was performed in R [28] using the R packages Dosefinding [29] and drc [30].

## Results

### Particle atmospheres

The average airborne BaSO_4_ concentration was 4.013 mg/m^3^ (± 1.8%) (target concentration: 4.0 mg/m^3^). A particle concentration of 9.5 µg/m^3^ (± 82.5%) was determined for the filtered air. The particle size distribution at 4.0 mg/m^3^ was monomodal with a relatively small geometric standard deviation of 1.50 and yielded a median aerodynamic diameter of 1.9 µm (± 2.1%). On average, 1130 particles per cm^3^ were measured.

### Questionnaire

The evaluation of parts of the questionnaires relevant for this study (feeling of fever, feeling sick, muscle pain, throat irritation and/or cough) did not demonstrate an increase of the effect rating after BaSO_4_ exposure compared to sham. On average, the relative symptom sum score for all questions at each time point was 4.6%. The highest relative sum score of 7.5% was found for “throat irritation and/ or cough” 22 h after BaSO_4_ exposure. No significant differences were observed between BaSO_4_ and sham exposures. When asked, the subjects could not distinguish the different exposure scenarios.

### Body temperature and other clinical features

Nearly all circadian temperature fluctuations were inside 1.3 °C and lower than 37.5 °C. Except for one female subject who measured an increased temperature of 37.8 °C in the evening after her BaSO_4_ exposure, but without reporting any symptoms, no increase of body temperature (≥ 37.5 °C) was observed after both exposure scenarios (Additional file 1: Figure S3). The median temperatures did not differ significantly when comparing sham and BaSO_4_ exposures at each time point. The detected minimum in the second half of the night reflected a physiological fluctuation in the temperature course of a day. Normally, the maximum body temperature is expected in the afternoon [31]. In the subjects of this study, it was in the morning, which may be due to the physical exertion on the ergometer during the exposures, which always took place in the morning.

No study participant showed clinical signs after the BaSO_4_ exposures, which had also no effects on blood pressure, FeNO and all lung function parameters (data not shown).

### Blood and sputum parameters

Table 2 shows the univariate evaluation of the time course of blood parameters (leucocytes, neutrophils, lymphocytes, monocytes, thrombocytes, CRP, SAA) and sputum parameters (IL-8, MMP-9, TIMP-1, total protein, total cell number, neutrophils) of our BaSO_4_ study. All blood parameters showed no significant changes when comparing the values before and 22 h after sham and BaSO_4_ exposure, respectively. Furthermore, the obtained values from five examinations without BaSO_4_ exposure (one baseline examination, two examinations before sham and BaSO_4_ exposure, one examination 22 h after sham exposure and one final examination) were not significantly different one from another. In addition, all sputum parameters were unaffected by BaSO_4_ exposure and showed no significant differences compared to sham exposures. Univariate calculations could be confirmed with multivariate generalized estimating equations (GEE) logistic regression and also showed no significant differences in blood and sputum parameters (data not shown).

**Table 2:** Acute effects of BaSO_4_ (4 mg/m^3^) or sham (0 mg/m^3^) exposure on blood and induced sputum parameters at different time points [median (minimum–maximum)]

Parameters	Baseline test	BaSO_4_ [mg/m^3^]	Directly before exposure	22 h after exposure	Final examination
**Blood**					
Leukocytes [1/nL]	5.50 (4.60–8.50)	0	6.25 (4.80–13.10)	5.60 (4.30–9.50)	6.20 (4.50–9.20)
		4	5.45 (4.00–8.40)	6.25 (4.60–13.90)	
Neutrophils [%]	56 (40.8–70)	0	57.5 (38–76)	60.5 (41–69)	57 (41–72)
		4	55 (41–73)	56 (48–80)	
Neutrophils [1/nL]	3.08 (2.21–5.94)	0	3.69 (2.00–9.94)	3.47 (1.76–6.50)	3.63 (1.87–6.64)
		4	3.25 (1.75–6.07)	3.42 (2.42–11.10)	
Lymphocytes [%]	29 (22–40)	0	30 (14–45)	29 (19–44)	32 (19–45)
		4	32 (18–47)	31.5 (11–38)	
Lymphocytes [1/nL]	1.76 (1.37–2.28)	0	1.86 (1.59–2.49)	1.83 (1.14–2.44)	1.95 (1.45–2.73)
		4	1.69 (1.16–2.79)	1.81 (0.92–2.82)	
Monocytes [%]	9 (5–12)	0	8.5 (4–12)	8.5 (5–12)	8.5 (5–11)
		4	7.5 (5–12)	8 (5–12)	
Monocytes [1/nL]	0.53 (0.34–0.81)	0	0.53 (0.39–0.82)	0.55 (0.3–0.72)	0.47 (0.33–0.82)
		4	0.47 (0.30–0.65)	0.50 (0.31–1.00)	
Thrombocytes [1/nL]	242 (192–346)	0	253 (181–339)	238 (168–339)	231 (176–339)
		4	226 (172–344)	231 (178–326)	
Erythrocytes [1/nL]	4.75 (4.10–5.35)	0	4.80 (4.10–5.50)	4.75 (4.10–5.40)	4.80 (4.00–5.30)
		4	4.75 (4.10–5.60)	4.85 (3.90–5.50)	
CRP [mg/L]	0.78 (0.06–4.24)	0	0.92 (0.08–3.63)	1.05 (0.04–4.73)	0.89 (0.00–7.12)
		4	0.86 (0.06–7.33)	1.14 (0.00–5.43)	
SAA [µg/L]	10,873 (< 1880–51,897)	0	15,366 (3262–87,932)	18,379 (3607–256,193)	11,082 (< 1880–158,188)
		4	8674 (2381–46,633)	10,318 (< 1880–107,439)	
**Induced sputum**					
IL-8 [ng/L]	1771 (193–12,768)	0	–	684 (164–16,177)	1303 (219–12,775)
		4	–	1128 (166–36,918)	
MMP-9 [µg/L]	161 (33–852)	0	–	61 (15–764)	58 (13–335)
		4	–	104 (20–1329)	
TIMP-1 [µg/L]	9.27 (0.82–41.40)	0	–	3.26 (1.01–30.12)	2.65 (0.57–21.65)
		4	–	4.62 (0.91–54.50)	
Total protein [mg/L]	260 (76–846)	0	–	189 (66–698)	146 (67–477)
		4	–	197 (59–931)	
Total cell number [× 10^5^]	60.33 (5.00–108.41)	0	–	29.63 (8.55–212.44)	28.60 (7.33–62.73)
		4	–	35.10 (8.86–140.10)	
Neutrophils [%]	8.75 (0–34)	0	–	1.5 (0–40)	3 (0–28)
		4	–	1.25 (0–32.5)	
Neutrophils [× 10^4^]	37.85 (0–368.61)	0	–	5.13 (0–484.59)	8.2 (0–133)
		4	–	5.62 (0–455.33)	

As an exception, leukocytes [1/nL] and monocytes [1/nL] showed significant differences between measurements directly before both exposure scenarios. However, these findings were not affected by the BaSO_4_ exposure. Additionally GEE logistic regression detected a significant difference between before and after exposure to BaSO_4_ for monocytes [1/nL]. With regard to the above-mentioned significance without exposure effect and the univariate calculations, these effects were considered biologically/toxicologically not relevant.

### Calculation of deposition rates of inhaled BaSO_4_ particles

**Fig. 2:**
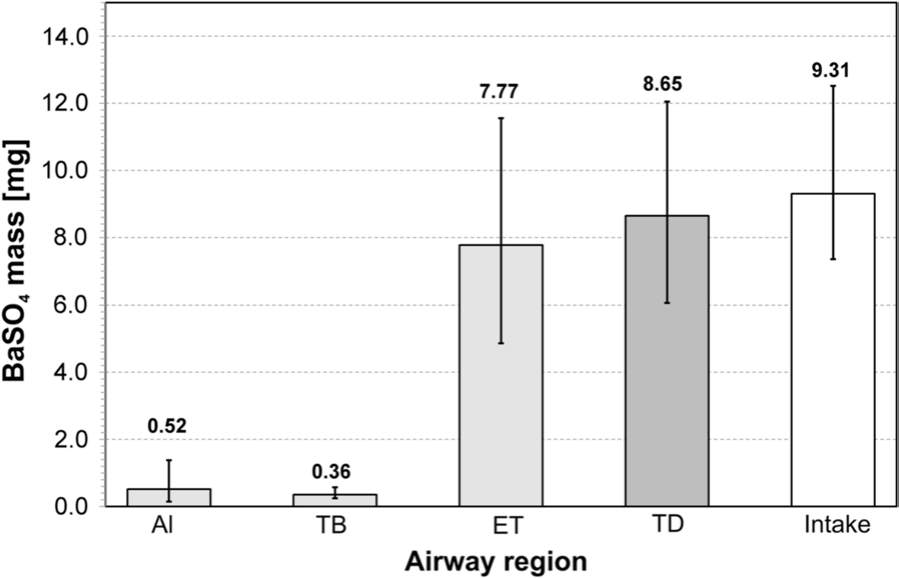
Calculated lung deposition efficiency of BaSO_4_ particles in dependence of airway regions (Al: alveolar region, TB: tracheobronchial region, ET: extrathoracic region) and particle size. TD: total deposition (sum of Al + TB + ET), Intake: Total inhaled mass of BaSO_4_ particles

Figure 2 shows the estimation of the masses of deposited BaSO_4_ particles of different airway regions (alveolar, tracheobronchial, and extrathoracic region) according to particle sizes. The mean values of the deposited masses of all participants are shown. The whiskers represent the minimum and maximum values. The total inhaled mass of BaSO_4_ particles is also shown (Intake). According to the ICRP-model, alveolar deposition rate (Al) was 5.6% and 3.9% of the particles were deposited in the tracheobronchial region (TB). Most of the particulate mass was deposited in the extrathoracic region (ET) and corresponded to a fraction of about 83.5%. Overall 93.0% of the total inhaled mass of BaSO_4_ particles was deposited in the airways, 7.0% of the mass was exhaled, respectively.

### Particle-induced cell migration assay

**Fig. 3:**
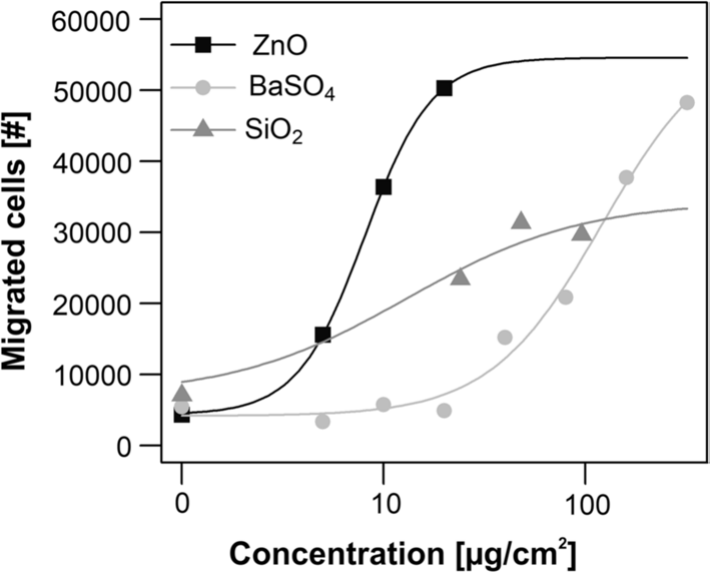
Chemotaxis (migrated cells) of the unexposed dHL-60 cells in response to NR8383 cell supernatants obtained from incubations with increasing compound concentrations. For comparison data of the ZnO, induced chemotaxis are shown. Commercially available silica (SiO_2_) nanoparticles were used as positive control that allows comparison with historical data. Results represent fitted dose–response models for the numbers of in vitro migrated cells after treatment with ZnO and BaSO_4_. Results represent arithmetic means and standard deviations of three independent experiments

Challenge of NR8383 cells with BaSO_4_ particles resulted in cell supernatants that had a weak chemotactic activity on dHL-60 cells at very high concentrations and were much weaker than the silica positive control. ZnO particles showed the strongest effects, even considerably stronger than silica (Fig. 3).

The small number of data points at each dose led to a high variation. Nevertheless, the model courses and especially the 50% effective concentrations (EC_50_) values indicate a good tendency for the real courses. The following EC_50_ with corresponding 95% confidence intervals were calculated for the different compounds based on the separately adjusted dose–response models: ZnO: 8.06 [2.55, 13.57], BaSO_4_: 121.20 [25.00, 217.41], SiO_2_: 13.06 [-12.20, 38.31] μg/cm^2^.

The four-parameter log-logistic model for ZnO shows a rapid steep sigmoidal curve. Additionally, the EC_50_ of ZnO with 8.06 and its confidence interval shows the highest activity of the investigated substances. Also the confidence interval of ZnO is entirely lower than that of BaSO_4_, which emphasizes the higher activity of ZnO.

## Discussion

The deviation of TLV is often based on animal experiments. It is desirable to support these derivations by human studies. However, the endpoints that can be examined and the study design are limited here by ethical considerations. For this purpose, it is necessary to identify sensitive and specific biological effect markers, which ideally allow a differentiation between "adverse" and "non-adverse “effects. For the acute BaSO_4_ inhalation study, we selected those parameters that had been shown to be sensitive for the detection of airway inflammation in our previous ZnO studies [11, 12, 18].

A controlled inhalation study with human volunteers at our exposure lab using nano-sized ZnO at concentrations up to 2 mg/m^3^ showed concentration-dependent systemic inflammatory effects, including an increase of body temperature in several subjects, as well as an increase in blood neutrophils, and acute phase protein levels [11]. Local effects were also detectable in induced sputum, but without a concentration-dependent relationship [18]. In an other ZnO study, subjects were exposed to nano- and micro-sized ZnO for 2 h at concentration levels of 2 mg/m^3^ each [12]. We could show that biological effects were more pronounced after exposure of micro-sized ZnO particles. According to the final investigations 2 to 6 weeks after exposures all acute effects were completely reversible in both studies. Furthermore, the results of these studies support the assumption that the observed ZnO effects were not caused by physical particle effects in the sense of a lung overload.

Criteria for setting our BaSO_4_ concentration were based on preliminary tests without subjects. They showed that atmospheres containing BaSO_4_ with concentrations above 6 mg/m^3^ become visible in the exposure unit and blinding of both exposures (BaSO_4_ and sham) would not be possible. Therefore, we set the maximum applicable concentration to 4.0 mg/m^3^ with a sufficient distance to 6 mg/m^3^ so that both exposure conditions were indistinguishable for the subjects. For organizational reasons, the exposure time was set at 2 h.

**Fig. 4:**
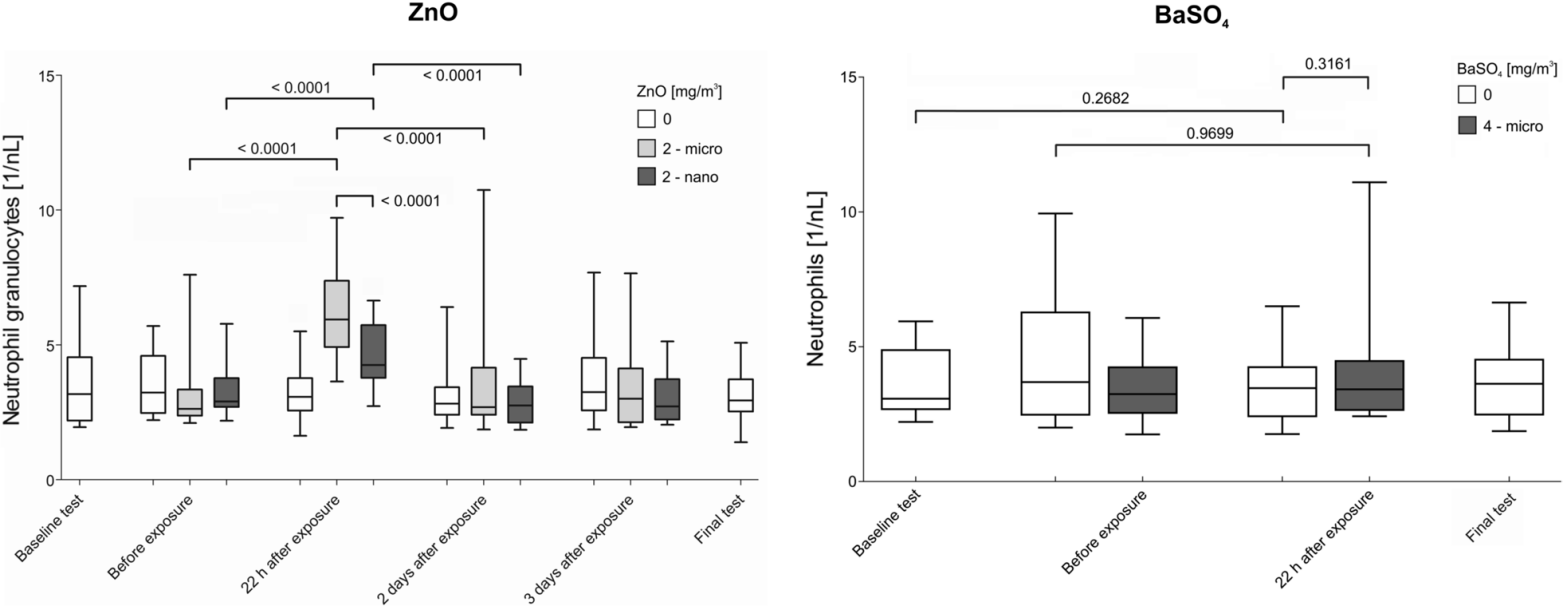
Time courses of neutrophils in blood in the second ZnO study [12] and the BaSO_4_ study

The most important result of the present study is the absence of any significant differences between BaSO_4_ and sham exposures in all parameters investigated. There was no evidence of local or systemic effects after inhalation of micro-sized BaSO_4_ at 4.0 mg/m^3^ for 2 h. Body temperature measurements and symptom questionnaires also showed no differences between both exposure scenarios. The study design was sufficiently sensitive to detect ZnO effects, but failed to reveal any effects in the case of micro-sized BaSO_4_. Figure 4 shows a comparison of neutrophils in blood from the second ZnO study and the present BaSO_4_ study.

ZnO effects may have been influenced by random fluctuations and measurement inaccuracies, which however, did not invalidate the significant changes in neutrophils in blood. For BaSO_4_, on the other hand, a similar scattering was measured, but no significant changes were detectable neither between different time points nor between BaSO_4_ and sham exposure. However, the scatterings were sufficiently small to rule out coincidental findings that would indicate a toxic property.

In contrast to this study, Sikkeland and co-workers found elevations in several parameters in induced sputum 24 h after aluminium oxide exposure (volume median diameter particle size 3.2 μm; 3.8–4.0 mg/m^3^ for 2 h) [10]. The blood parameters were measured 4 h after exposure, but did not show any changes and may have been overlooked: According to our experience, this time point may have been chosen too short, effects occurred significantly later [11, 12]. Other studies also suggest this [32, 33]. However, since the chemical purity and the chemical modification of the used material were unclear, it cannot be excluded that the observable effects were caused by non-inert containing substances that had a weak substance-specific toxicity.

Comparison of the calculations for the estimation of the deposition efficiencies between our second ZnO study [12] and the present study yielded a higher deposition ratio of BaSO_4_ in the airways. One explanation is that the mean particle size of BaSO_4_ with 1.90 µm is larger than that of the micro-sized ZnO with 1.33 µm and according to the ICRP model results in a higher deposition rate. This estimate is supported by the fact that BaSO_4_ did not cause any detectable effects even at more than twice the deposited mass (8.65 mg) compared to the mass of micro-sized ZnO (3.04 mg) used in the second ZnO study.

One possible weakness of the present study is the restriction of the recording of systemic and local effect parameters at specific time points. A possible absence of effects may be caused by an earlier or later increase of effect parameters compared to the sampling times. Furthermore, no investigations with nano-sized BaSO_4_ particles were performed in this study. Using BaSO_4_ nanoparticles, Molina et al. [8] and Keller et al. [9] have shown the dissolution and release of barium ions under physiological conditions. Therefore, it cannot be excluded that our findings does not apply to nano-sized BaSO_4_. However, this acute study is not suitable to be extrapolated to repeated or chronic inhalation in humans, since GBP are toxic only in case of lung overload and the doses chosen in this study were far below this level. No conclusions can be drawn about the extent of accumulation in case of repeated exposure, nor about the elimination kinetics of the deposited BaSO_4_ particles in the respiratory tract. An acute inhalation study in humans has limited evidence of transferability of data from animal studies to humans for chronic endpoints.

As demonstrated in our ZnO studies [11, 12], the strength of this study is the lack of effects after sham exposures (0 mg/m^3^ BaSO_4_). Many control conditions were performed without BaSO_4_ exposure (five control conditions for blood parameters: one baseline examination, two examinations before exposures, one examination 22 h after sham exposure and one final examination). For the sputum parameters three control scenarios (one baseline examination, one examination 22 h after sham exposure and one final examination) were available. Thus, accidental variabilities were minimized.

Since this study did not detect any differences of parameters between BaSO_4_ and sham exposures, the question arises whether recruitment of 16 volunteers is sufficient. Howevecell migration between 10 and 20r, we conducted this study with a similar design as the ZnO studies, where robust parameters showed clear changes after ZnO exposures. Since the study design was sensitive enough to show these acute ZnO effects, we can assume that it is also true for BaSO_4_ under the chosen exposure conditions.

In vitro data can supplement in vivo data in order to support mechanistic propositions. Therefore we confirmed the inert character of the BaSO_4_ in vitro. We used the particle-induced chemotaxis assay to do this (PICMA) [6]. This assay mimics the particle induced accumulation of neutrophils which is a hallmark of particle- induced inflammation. Polymorphonuclear neutrophil (PMN) count and total protein concentration are usually the most sensitive, valid parameters that indicate particle toxicity as well in animal experiments (see for example [34]). The assay is very sensitive down to the subtoxic range. However, in vitro studies can in general also be carried out at very high doses. In this study, BaSO_4_ causes a very slight increase in migrated cells between 100 and 200 µg/cm^2^, compared to ZnO that induces strong cell migration between 10 and 20 µg/cm^2^ (Additional file 1: Figure S4). Concordantly, BaSO_4_ of different particle sizes neither caused an inflammatory nor a cytotoxic response investigated by the PICMA, by the release of inflammatory mediators (CCL2, TNF-a, IL-6), by apoptosis or necrosis up to the highest tested dose each [7]. We used NR8383 rat alveolar macrophages for particle challenge to produce cell- and particle supernatants that attract d-HL-60 cells—a well established model cell line for neutrophils. Challenge of NR8383 cells with the BaSO_4_ particles that were used in the inhalation study yielded cell supernatants that acted very weakly chemotactic towards the dHL-60 cells at high doses. This effect was about 9 times less pronounced compared to the SiO_2_ positive control and about 15 times weaker than the ZnO nanoparticles, based on the calculated EC_50_ values. A comparison with toxic fibers helps to get an idea of the strength of the effect: ZnO induced chemotaxis of d-HL60 cells is approximately 10-times weaker than asbestos fibers and multiwalled carbon nanotubes [35]. It is likely that the high EC_50_ value of BaSO_4_ even underestimates this difference, since the curve does not reach a plateau, which normally leads to an underestimation of the calculated EC_50_ value. This is in contrast to the ZnO and SiO_2_ particles or other toxic particles or fibers and due to the fact that BaSO_4_ does not show any cytotoxicity (Additional file 1: Figure S5). We could not find any other biological or inflammatory in vitro effects in the literature, including elevation of inflammatory signaling molecules after in vitro challenge with very high doses of BaSO_4_ [7, 36], in contrast to the findings with toxic particles and fibers [35].

These in vitro data thus appear to mirror the data from the controlled human BaSO_4_ and ZnO inhalation studies, which showed systemic and local inflammatory effects, including elevation of blood neutrophils following inhalation of ZnO but not BaSO_4_. This comparison suggests that weak chemotaxis in vitro at such high particle concentrations is consistent with the assessment as a chemically inert particle.

Even if limitations of our study design have to be considered, in particular that only an acute study was performed, the results allow the conclusion that acute exposures at the said level rule out irritative effects with some degree of certainty. As similar inhalation studies with GBP were not available previously, such studies could not be used for the setting of a TLV. It is certainly difficult to transfer results from acute to chronic inhalation exposures to GBP. Longer or repeated inhalation studies in humans are too laborious and thus not considered a realistic option. This study should stipulate discussions in committees which recommend TLVs like the American Conference of Governmental Industrial Hygienists (ACGIH), the German MAK-commission, or others.

## Conclusions

In summary, there was no evidence of local or systemic effects after acute inhalation of micro-sized BaSO_4_ in humans at a concentration of 4.0 mg/m^3^ for 2 h. However, this study does not allow extrapolation to chronic exposures. The inhalation data in humans together with the particle-induced chemotaxis data in vitro study add support to the hypothesis that GBP are toxic under lung overload conditions which cannot be reached in acute human exposures. This study supports the results of animal experiments that micro-sized BaSO_4_ is an ‘inert’ particle, in contrast to ZnO.

## Supplementary Information

**Additional file 1**. **Figure S1.** Averaged particle size distribution of airborne BaSO_4_ particles and filtered air. **Figure S2.** SEM image of BaSO_4_ particles. **Figure S3.** 24 h temperature profile of all subjects. **Figure S4.** Chemotaxis (migrated cells) of the unexposed dHL-60 cells in response to NR8383 cell supernatants. **Figure S5.** Cytotoxicity of ZnO towards NR8383 cells according to the alamarBlueTM test.

## Data Availability

The datasets generated and/or analysed during the current study are not publicly available due to the very extensive data collection, but are available from the corresponding author on reasonable request.

## Abbreviations

EC_50_: 50% Effective concentration
APS: Aerosol particle sizer
ACGIH: American Conference of Governmental Industrial Hygienists
BaSO_4_: Barium sulfate
BMI: Body mass index
BET: Brunauer Emmet Teller
CCL2: CC-chemokine ligand 2
CRP: C-reactive protein
CI: Confidence intervals
ECG: Electrocardiogram
FeNO: Fractional exhaled nitric oxide
GEE: Generalized estimating equations
GBP: Granular biopersistent particles
IL-8: Interleukin-8
IL-6: Interleukin-6
ICRP: International Committee for Radiological Protection
MMP-9: Matrix metalloproteinase-9
NO: Nitric oxide
OEL: Occupational exposure limit
OR: Odds ratios
SpO2: Oxygen saturation
PMN: Polymorphonuclear neutrophil
PSG: Polysomnography
SEM: Scanning electron microscopy
SAA: Serum amyloide A
SiO_2_: Silica
TEOM: Tapered elemental oscillating microbalance
TLV: Threshold limit value
TIMP-1: Tissue inhibitors of metalloproteinases-1
TNF-a: Tumor necrosis factor alpha
ZnO: Zinc oxide

## References

[1] DFG. The MAK collection for occupational health and safety, Online ISBN: 9783527600410, 53th edn. Wiley VCH Weinheim 2011.

[2] European Center for Ecotoxicology and Toxicology of Chemicals (ECETOC). Technical Report No. 122. Poorly soluble particles, lung overload. ISSN-0773-8072-122 (print), ISSN-2079-1526-122 (online). 2013.

[3] TRGS 900, Technische Regeln für Gefahrstoffe, Arbeitsplatzgrenzwerte (Fassung 31.1.2018). Ausschuss für Gefahrstoffe - AGS-Geschäftsführung - BAuA, Ausgabe: Januar 2006, BArBl Heft 1/2006 S. 41–55, zuletzt berichtigt: GMBl 2021, S. 580 [Nr. 25] (vom 23.04.2021)

[4] Konduru N, Keller J, Ma-Hock L, Gröters S, Landsiedel R, Donaghey TC, Brain JD, Wohlleben W, Molina RM (2014). Biokinetics and effects of barium sulfate nanoparticles. Part Fibre Toxicol.

[5] DFG. MAK value documentation for barium sulfate dust. 10.1002/3527600418.mb772743stae6217. Wiley VCH Weinheim 2017.

[6] Westphal GA, Schremmer I, Rostek A, Loza K, Rosenkranz N, Brüning T, Epple M, Bünger J (2015). Particle-induced cell migration assay (PICMA): a new in vitro assay for inflammatory particle effects based on permanent cell lines. Toxicol In Vitro.

[7] Loza K, Föhring I, Bünger J, Westphal GA, Köller M, Epple M, Sengstock C (2016). Barium sulfate micro- and nanoparticles as bioinert reference material in particle toxicology. Nanotoxicology.

[8] Molina RM, Konduru NV, Queiroz PM, Figueroa B, Fu D, Ma-Hock L, Groeters S, Schaudien D, Brain JD (2019). Fate of barium sulfate nanoparticles deposited in the lungs of rats. Sci Rep.

[9] Keller JG, Graham UM, Koltermann-Jülly J, Gelein R, Ma-Hock L, Landsiedel R, Wiemann M, Oberdörster G, Elder A, Wohlleben W (2020). Predicting dissolution and transformation of inhaled nanoparticles in the lung using abiotic flow cells: the case of barium sulfate. Sci Rep.

[10] Sikkeland LIB, Alexis NE, Fry RC, Martin E, Danielsen TE, Søstrand P, Kongerud J (2016). Inflammation in induced sputum after aluminium oxide exposure: an experimental chamber study. Occup Environ Med.

[11] Monsé C, Hagemeyer O, Raulf M, Jettkant B, van Kampen V, Kendzia B, Gering V, Kappert G, Weiss T, Ulrich N, Marek E-M, Bünger J, Brüning T, Merget R (2018). Concentration-dependent systemic response after inhalation of nano-sized zinc oxide particles in human volunteers. Part Fibre Toxicol.

[12] Monsé C, Raulf M, Jettkant B, van Kampen V, Kendzia B, Schürmeyer L, Seifert CE, E-M Marek, Westphal G, Rosenkranz N, Merget R, Brüning T, Bünger J. Health efects after inhalation of micro- and nano-sized zinc oxide particles in human volunteers. Arch Tox. 2021;95:53–65. 10.1007/s00204-020-02923-y PMC7811523 33001223

[13] Monsé C, Sucker K, van Thriel C, Broding HC, Jettkant B, Berresheim H, Wiethege T, Käfferlein H, Merget R, Bünger J, Brüning T (2012). Considerations for the design and technical setup of a human whole-body exposure chamber. Inhal Toxicol.

[14] Wohlleben W, Mielke J, Bianchin A, Ghanem A, Freiberger H, Rauscher H, Gemeinert M, Hodoroaba V-D (2017). Reliable nanomaterial classification of powders using the volume-specific surface area method. J Nanopart Res.

[15] Merget R, Heinze E, Neumann L, Taeger D, Brüning T. Comparison of the Pari Provotest II reservoir- and the ATS dosimeter method for the assessment of bronchial hyperresponsiveness. In: Bruening T, Harth V, Zaghow M (Hrsg.): Proceedings of the 45th annual meeting of the German Society for Occupational and Environmental Medicine. Gentner Publishing Company, Stuttgart: 2005;624–625.

[16] Raulf M, Hoffmeyer F, van Kampen V, Deckert A, Brüning T, Bünger J (2015). Cellular and soluble inflammatory markers in induced sputum of composting plant workers. Adv Exp Med Biol.

[17] Raulf M, van Kampen V, Neumann HD, Liebers V, Deckert A, Brüning T, Bünger J, Hoffmeyer F (2017). Airway and blood inflammatory markers in waste collectors. Adv Exp Med Biol.

[18] Monsé C, Raulf M, Hagemeyer O, van Kampen V, Kendzia B, Gering V, Marek E-M, Jettkant B, Bünger J, Merget R, Brüning T (2019). Airway inflammation after inhalation of nano-sized zinc oxide particles in human volunteers. BMC Pulm Med.

[19] American Thoracic Society and European Respiratory Society (ATS/ERS). Recommendations for standardized procedures for the online and offline measurement of exhaled lower respiratory nitric oxide and nasal nitric oxide. Am J Respir Crit Care Med. 2005;171:912–930. 10.1164/rccm.200406-710ST 15817806

[20] Criée CP, Sorichter S, Smith HJ, Kardos P, Merget R, Heise D, Berdel D, Köhler D, Magnussen H, Marek W, Mitfessel H, Rasche K, Rolke M, Worth H, Jörres RA (2011). Bodyplethysmography - its principles and clinical use. Respir Med.

[21] American Thoracic Society (ATS). Standardization of spirometry, 1994 Update (1995) Am J Respir Crit Care Med. 1995;152:1107–1136. 10.1164/ajrccm.152.3.7663792 7663792

[22] Bonferroni CE. Teoria statistica delle classi e calcolo delle probabilità. Pubblicazioni del R Istituto Superiore di Scienze Economiche e Commerciali di Firenze. 1936;8:3–62.

[23] Liang K-Y, Zeger SL (1986). Longitudinal data analysis using generalized linear models. Biometrika.

[24] O’Connell MA, Belanger BA, Haaland PD (1993). Calibration and assay development using the four-parameter logistic model. Chemometr Intell Lab Syst.

[25] Pinheiro JC, Bretz F, Branson M. Analysis of dose-response studies - Modeling approaches. Ting N, ed. Dose finding in drug development. New York: Springer; 2006.

[26] Guha S, Hariharan P, Myers MR (2014). Enhancement of ICRP's lung deposition model for pathogenic bioaerosols. Aerosol Sci Tech.

[27] ICRP Human respiratory tract model for radiological protection. ICRP Publication 66. Ann. ICRP;24,1–3. 1994. 7726471

[28] R Core Team: A language and environment for statistical computing. R Foundation for Statistical Computing, Vienna, Austria 2021. https://www.R-project.org/

[29] Bornkamp B (2019). DoseFinding: Planning and analyzing dose finding experiments. R package version 0.9-17. https://CRAN.R-project.org/package=DoseFinding

[30] Ritz C, Baty F, Streibig JC, Gerhard D. Dose-response analysis using R. PLOS One. 2015;10(12), e0146021. 10.1371/journal.pone.0146021 PMC4696819 26717316

[31] Refinetti R, Menaker M (1992). The circadian rhythm of body temperature. Physiol Behav.

[32] Brand P, Bauer M, Gube M, Lenz K, Reisgen U, Spiegel-Ciobanu VE, Kraus T (2014). Relationship between welding fume concentration and systemic inflammation after controlled exposure of human subjects with welding fumes from metal inert gas brazing of zinc-coated materials. J Occup Environ Med.

[33] Baumann R, Joraslafsky S, Markert A, Rack I, Davatgarbenam S, Kossack V, Gerhards B, Kraus T, Brand P, Gube M (2016). IL-6, a central acute-phase mediator, as an early biomarker for exposure to zinc-based metal fumes. Toxicology.

[34] Landsiedel R, Ma-Hock L, Hofmann T, Wiemann M, Strauss V, Treumann S, Wohlleben W, Gröters S, Wiench K, van Ravenzwaay B (2014). Application of short-term inhalation studies to assess the inhalation toxicity of nanomaterials. Part Fibre Toxicol.

[35] Westphal GA, Rosenkranz N, Brik A, Weber D, Föhring I, Monsé C, Kaiser N, Hellack B, Mattenklott M, Brüning T, JohnenG, Bünger J. Multi-walled carbon nano tubes induce stronger migration of inflammatory cells in vitro than asbestos or granular particles but a similar pattern of inflammatory mediators. Toxicol In Vitro. 2019;58:215–223. 10.1016/j.tiv.2019.03.036 30928694

[36] Schremmer I, Brik A, Weber DG, Rosenkranz N, Rostek A, Loza K, Brüning T, Johnen G, Epple M, Bünger J, Westphal GA (2016). Kinetics of chemotaxis; cytokine; and chemokine release of NR8383 macrophages after exposure to inflammatory and inert granular insoluble particles. Toxicol Lett.

