# Supplementary material for: No inflammatory effects after acute inhalation of barium sulfate particles in human volunteers
Source: BMC Pulm Med. 2022 Jun 16;22:233. doi: 10.1186/s12890-022-02021-y (PMC9205122; doi:10.1186/s12890-022-02021-y)
Supplement: Supplementary file 1 — Additional file 1. Figure S1. Averaged particle size distribution of airborne BaSO4 particles and filtered air. Figure S2. SEM image of BaSO4 particles. Figure S3. 24 h temperature profile of all subjects. Figure S4. Chemotaxis (migrated cells) of the unexposed dHL-60 cells in response to NR8383 cell supernatants. Figure S5. Cytotoxicity of ZnO towards NR8383 cells according to the alamarBlueTM test. [file 12890_2022_2021_MOESM1_ESM.docx]

**No inflammatory effects after acute inhalation of barium sulfate particles in human volunteers**

**BMC Pulmonary Medicine**

Christian Monsé, Götz Westphal, Monika Raulf, Birger Jettkant, Vera van Kampen, Benjamin Kendzia, Leonie Schürmeyer, Christoph Edzard Seifert, Eike-Maximilian Marek, Felicitas Wiegand, Christopher Wegener, Nina Rosenkranz, Rolf Merget, Thomas Brüning, Jürgen Bünger

Institute for Prevention and Occupational Medicine of the German Social Accident Insurance, Institute of the Ruhr University Bochum (IPA), Bürkle-de-la-Camp-Platz 1, 44789 Bochum, Germany

1. **Characterization of BaSO_4_ particles**

Figure S1 shows the particle size distributions of BaSO_4_ particles at target concentrations of 4.0 and 0 mg/m^3^.





**Figure S1:** Averaged particle size distribution of airborne BaSO_4_ particles at 4.0 mg/m^3^ (solid line) and filtered air at 0 mg/m^3^ (rigged line). In addition, the error bars of each individual size channel are shown. The error bars of the sham exposure condition are not visible due to the small magnitude.

The curves represent an average of all days of the 2 h exposures at 4.0 and 0.0 mg/m^3^, respectively. The particle size distribution at 4.0 mg/m^3^ was monomodal with a relatively small geometric standard deviation of 1.50 and yielded a median aerodynamic diameter of 1.9 µm (+/- 2.1 %). On average, 1130 particles per cm^3^ were measured. The APS was equipped with a 1:20 aerosol diluter.

Figure S2 shows the micro-sized BaSO_4_ particles which consisted of individual crystals and which are rounded at their edges. These particles were taken directly from the chemical packaging.


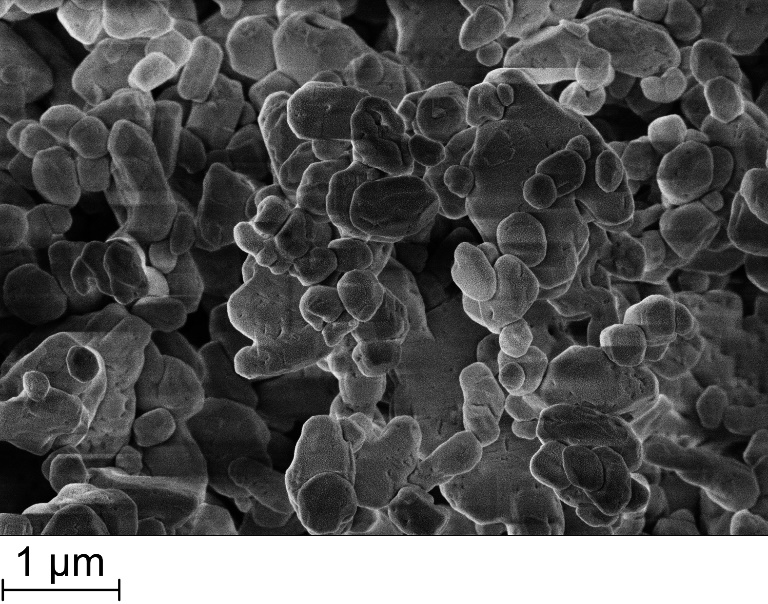


**Figure S2:** SEM (scanning electron microscopy) image of BaSO_4_ particles.

1. **Body temperature measurements**

**

**

**Figure S3:** 24 h temperature profile of all subjects, including before, during, and after exposures with filtered air and BaSO_4_. White box plots: sham exposure, grey box plots: BaSO_4_ exposure.

1. **Further *In vitro* experiments**

**Figure S4:** Chemotaxis (migrated cells) of the unexposed dHL-60 cells in response to NR8383 cell supernatants that were obtained from incubation with ZnO particles in concentrations between 1.25 and 80.0 µg/cm^2^. Data represent mean ± SD (n = 3). Commercially available silica nanoparticles served as positive control.

**Figure S5:** Cytotoxicity of ZnO towards NR8383 cells according to the alamarBlue^TM^ test, a fluorometric assay in which the metabolic activity of the cells is determined. The cytotoxicity is plotted as absolute fluorescence versus ZnO concentrations between 1.0 and 128.0 µg/cm^2^. Data are expressed as mean ± SD (n = 3). The alamarBlue^TM^ test measures the reduction of the blue, non-fluorescent resazurin into a highly fluorescent red colour.

The alamarBlue^TM^ test was carried out according to the procedure specified by the manufacturer. In short: The cells are sown in a defined number of cells in a multiwell plate and incubated for 24 hours in an incubator (37 °C, 5 % CO_2_). Then the particles are added in defined dilutions. Following another incubation for 24 hours the detection reagent is added. The reduction of the resazurin is determined photometrically.
